# Rapid increase of *Plasmodium falciparum dhfr/dhps *resistant haplotypes, after the adoption of sulphadoxine-pyrimethamine as first line treatment in 2002, in southern Mozambique

**DOI:** 10.1186/1475-2875-7-115

**Published:** 2008-07-01

**Authors:** Sonia Enosse, Pascal Magnussen, Fatima Abacassamo, Xavier Gómez-Olivé, Anita M Rønn, Ricardo Thompson, Michael Alifrangis

**Affiliations:** 1 Instituto Nacional de Saúde, Ministério de Saúde, Maputo, Mozambique; 2 Centro de Investigação em Saúde da Manhiça, Ministério de Saúde, Maputo, Mozambique; 3 DBL-Centre of Health Research and Development, Faculty of Life Sciences, Copenhagen University, Denmark; 4 MRC/Wits University, Rural Public Health and Health Transitions Research Unit (Agincourt), School of Public Health, University of the Witwatersrand, South Africa; 5 Centre for Medical Parasitology at Department of International Health, Immunology and Microbiology, University of Copenhagen and at Department of Infectious Diseases, Copenhagen University Hospital (Rigshospitalet), Denmark

## Abstract

**Background:**

In late 2002, the health authorities of Mozambique implemented sulphadoxine-pyrimethamine (SP)/amodiaquine (AQ) as first-line treatment against uncomplicated falciparum malaria. In 2004, this has been altered to SP/artesunate in line with WHO recommendations of using Artemisinin Combination Therapies (ACTs), despite the fact that all the neighbouring countries have abandoned SP-drug combinations due to high levels of SP drug resistance. In the study area, one year prior to the change to SP/AQ, SP alone was used to treat uncomplicated malaria cases. The study described here investigated the immediate impact of the change to SP on the frequency of SP and CQ resistance-related haplotypes in the *Plasmodium falciparum *genes *Pfdhfr, Pfdhps *and *Pfcrt *before and a year after the introduction of SP.

**Methods:**

Samples were collected during two cross sectional surveys in early 2002 and 2003 involving 796 and 692 children one year or older and adults randomly selected living in Maciana, an area located in Manhiça district, Southern Mozambique. Out of these, 171 and 173 *P. falciparum *positive samples were randomly selected to measure the frequency of resistance- related haplotypes in *Pfdhfr, Pfdhps *and *Pfcrt *based on results obtained by nested PCR followed by sequence-specific oligonucleotide probe (SSOP)-ELISA.

**Results:**

The frequency of the SP-resistance associated *Pfdhps *double mutant (SGEAA) haplotype increased significantly from 14% to 35% (P < 0.001), while the triple mutant *Pfdhfr *haplotype (CIRNI) remained high and only changed marginally from 46% to 53% (P = 0.405) after one year with SP as first-line treatment in the study area. Conversely, the combined *Pfdhfr/Pfdhps *quintuple mutant haplotype increased from 8% to 26% (P = 0.005). The frequency of the chloroquine resistance associated *Pfcrt*-CVIET haplotype was above 90% in both years.

**Conclusion:**

These retrospective findings add to the general concern on the lifespan of the combination of SP/artesunate in Mozambique. The high frequency of quintuple *Pfdhfr*/*Pfdhps *haplotypes found here as early as 2002 most likely cause ideal conditions for the development of artesunate tolerance in the *P. falciparum *populations and may even endanger the sensitivity to the second-line drug, Coartem^®^.

## Background

Large scale prevalence of *Plasmodium falciparum *chloroquine (CQ), amodiaquine (AQ) and sulphadoxine/pyrimethamine (SP) resistance has forced countries with endemic malaria to change to the artemisinin combination therapies (ACTs) as first-line treatment against uncomplicated malaria. The ACT drugs, currently being implemented in sub-Saharan Africa combine various artemisinin analogues with novel drugs (lumefantrine, i.e. Coartem^®^) or with already widely used drugs such as AQ and SP. ACTs are highly efficacious and are likely to decrease the rate of development of resistance, and thus expand the useful lifetime of the drugs [1]. Artemisinin derivates are acting and are eliminated rapidly after treatment and the development of resistance to ACTs will probably depend on the already existing background level of resistance to the partner drugs in the parasite population, as it has been observed in areas with high levels of resistance to SP and AQ [2,3]. Currently, SP combined with artesunate (ART) is the first-line treatment policy in few African countries, such as Mozambique (from 2004) and Northern Sudan [4,5]. In Mozambique, prior to adoption of the ACT strategy, the combination of AQ and SP as the first-line treatment was implemented in late 2002 following reports of high levels of chloroquine (CQ) resistance [6]. However, one year prior to this change, SP alone was used to treat uncomplicated malaria cases in the study area. Additionally, SP has recently been used in programmes examining the efficacy of intermittent presumptive treatment (IPT) intervention in pregnant women and infants in Mozambique [7,8].

The prevalence of combinations of single nucleotide polymorphisms (SNP) in the *P. falciparum *dihydrofolate reductase (*Pfdhfr*) and dihydropteroate synthetase (*Pfdhps*) genes have been correlated with resistance to SP *in vivo *[9,10]. In Africa, the *Pfdhfr *triple mutation, N51I+C59R+S108N, with wildtype at codons 50 (C50) and 164 (I164), is combined into a highly prevalent and resistant haplotype, CIRNI. For *Pfdhps *the double mutation, A437G + K540E, with wildtype at codons 436 (S436) 581 (A581) and 613 (A613) are combined into the resistant haplotype SGEAA. The combination of the two; CIRNI-SGEAA, have been suggested as a molecular marker of *in vivo *resistance [10]. However, recent studies have questioned the association between this combination of molecular markers and drug resistance *in vivo *by showing that differences in responses to SP treatment was a consequence of acquired immunity rather than parasite factors [11,12].

Resistance to both CQ and AQ is mainly associated with a single K76T mutation in the *P. falciparum *chloroquine resistance transporter gene (*Pfcrt*), but are probably involving mutations in the *P. falciparum *multi-drug resistance (*Pfmdr1*) gene as well [13,14]. Three main haplotypes in codons 72–76 of the *Pfcrt *gene exist, namely the wild type CVMNK and the CQ resistant haplotypes, CVIET and SVMNT [15], where CVIET is by far the most dominant mutant haplotype in Africa while SVMNT haplotypes has only been observed sporadically [16].

The objective of this study was to determine the frequency of *Pfdhfr*, *Pfdhps *and *Pfcrt *resistance related haplotypes in *P. falciparum *isolates collected before and a year after SP was introduced as first-line treatment, as replacement for CQ in the study area. Moreover, since Mozambique was implementing a treatment policy with SP in combination with artesunate as first line treatment of uncomplicated malaria, the findings of this study in addition to recent studies [17,18] is providing crucial baseline information of the level of drug resistance related haplotypes present in the parasite population prior to the adoption of the ACT strategy in the country.

## Methods

### Study population and samples

The study was conducted in Maciana, a village located in Manhiça district Southern Mozambique with 14.496 inhabitants [19]. The climate is characterized by a warm and wet season from November to April and a dry and cold season from May to October. The area is hyper-endemic for malaria and the transmission is perennial with marked seasonality and the majority of cases occur during the rainy season reaching a peak in during February and May. Ninety percent of all infections are caused by *P. falciparum*, but *P. malariae *and *P. ovale *are also observed. The population is mainly made up of farmers growing maize and beans and do have access to one health post which serves only outpatients [19]. Samples for analysis of molecular markers of drug resistance were collected during two cross sectional surveys involving 796 and 692 children one year or older and adults randomly selected from the Manhiça demographic surveillance system. The first cross-sectional study was conducted before the introduction of SP as first-line treatment in the study area, from December 2001 to January 2002 (survey 1); and the second, from January to February 2003 (survey 2). Finger prick blood samples were obtained for thick and thin blood smears and for filter paper blood spots.

Ethical clearance for the study was provided by the National Institute of Health, Ministry of Health of Mozambique Ethical Committee. The Danish National Committee on Biomedical Research Ethics commented on the proposal and recommended the study. Written informed consent was obtained from adults and the parents/guardians of the children before enrolment.

### DNA extraction and detection of polymorphisms in of *Pfdhfr*, *Pfdhps *and the *Pfcrt*

For practical reasons, 171 and 173 samples from cross sectional survey 1 and 2, respectively, were selected randomly for the analyses of polymorphisms in *Pfdhfr*, *Pfdhps *and *Pfcrt*. DNA extraction from bloodspots and nested PCR followed by the sequence specific oligonucleotide probe (SSOP)-ELISA was performed and analysed as previously described [20] using SSOPs targeting SNPs in *Pfdhfr *at c50/51 (CI/CN), c59 (C/R), c108 (S/N/T) and c164 (I/L) and in *Pfdhps *at c436/437 (AA/AG/SA/SG/FG) c540 (K/E), c581 (A/G) and c613 (A/S). The resulting single nucleotide polymorphisms (SNPs) were constructed into haplotypes. For *Pfcrt*, SSOPs targeting the haplotypes CVMNK, CVIET and SVMNT was used [20].

### Statistics

All statistical analyses were performed using SigmaStat software package version 3.0.1 and SPSS statistical package version 15.0. For the statistical analyses of the characteristics of the individuals sampled during the cross-sectional studies, the differences between groups were assessed using the Chi-square test for proportions and the Student t-test for continuous normal distributed variables. Frequencies of mutations between groups were compared using the Chi-square or Fisher exact test. In addition, a logistic regression model was used to compare haplotype distributions obtained in the two cross-sectional surveys while adjusting for the potential confounding effects of *P. falciparum *density and age.

## Results

A total of 244 and 192 samples were *P. falciparum *positive by microscopy in cross-sectional survey 1 and 2, respectively out of which 171 and 173 samples were chosen randomly for the genotyping analysis. The populations in the two surveys differed in respect to age and parasite density: In survey 2, the median age was significantly lower (P = 0.013) while the parasite density was significantly higher (P < 0.001) compared to survey 1 (Table 1). Only *Pfdhfr *wildtypes at c164 was found, while for *Pfdhps*, only wildtypes were observed at c518 and c613.

**Table 1:** Characteristics of the randomly selected samples for the analysis of molecular markers of drug resistance from cross sectional surveys in December 2001 (Cross1) and January 2003 (Cross 2)

	Cross1 N = 171	Cross2 N = 173	P-value
Median age (years) (25–75% percentile)	14 (8.0–27.8)	11 (6.0–23.5)	0.013
Gender, male (%)	43.3	45.7	0.772
Median *P. falciparum *density, parasites/μl (25–75% percentile)	125 (63–423)	332 (84–2242)	< 0.001
Donors with temperature >37.5°C (%)	4.1	4.6	0.982

P-values: Mann-Whitney Rank Sum Test or χ^2^-tests comparisons between the two cross sectional surveys.

**Figure 1:**
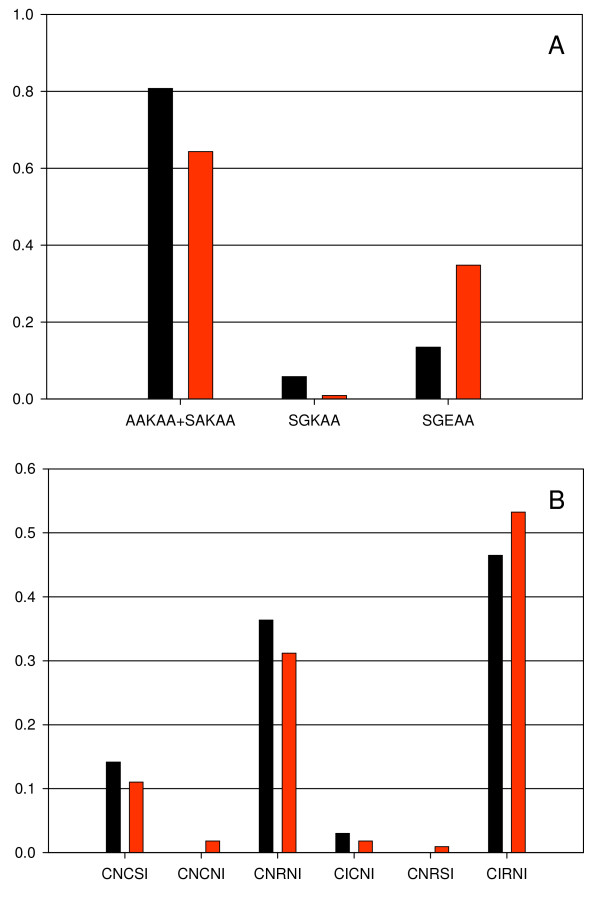
**The Frequency of *Pfdhps *(A) and *Pfdhfr *(B) haplotypes in December 2001 (black bars) and January 2003 (red bars) in Manhiça district**. After removing the PCR negatives and the samples with mixed haplotype infections (where a major haplotype could not be determined), for *Pfdhps*, N = 104 and 115 and for *Pfdhfr*, N = 99 and 109 from survey 1 and 2, respectively.

**Figure 2:**
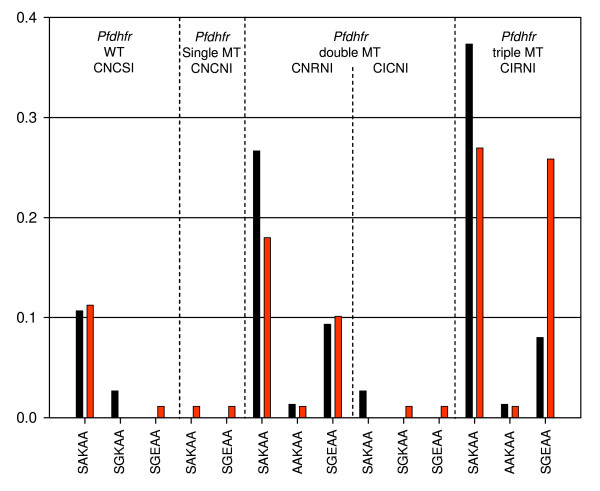
**The Frequency of combined *Pfdhfr/dhps *haplotypes in December 2001 (dark bars) and January 2003 (red bars) in Manhiça district in Manhiça district**. After removing the PCR negatives and the samples with mixed haplotype infections (where a major haplotype could not be determined), N = 75 and 89 samples from survey 1 and 2, respectively.

A significant increase in the frequency of *Pfdhps *double mutant haplotype, SGEAA from 13.5% to 34.8% over a year was found (*χ*^2 ^= 12.24, P < 0.001), mainly decreasing the frequency of *Pfdhps *wildtypes SAKAA/AAKAA from 80.8% to 64.3% (*χ*^2 ^= 5.893, P = 0.015; Figure 1A). For *Pfdhfr*, there was only a marginal difference between the two surveys with a frequency of the triple mutant haplotype, CIRNI at 46.5% and 53.2% in survey 1 and 2, respectively (*χ*^2 ^= 0.70, P = 0.405; Figure 1B). When combining the two loci, the frequency of parasites carrying the highly resistant *Pfdhfr/Pfdhps *quintuple haplotype increased from 8.0% to 25.8% (*χ*^2 ^= 7.78, P = 0.005) between survey 1 and 2. The change in frequency was not at the expense of the wildtype *Pfdhfr*/*Pfdhps *haplotype, CNCSI-SAKAA that remained stable at 11%, but rather on the double mutant haplotypes, where the CNRNI-SAKAA accounted for a decrease from 26.7% to 18.0% (*χ*^2 ^= 1.32, P = 0.250) and the triple mutant haplotype, mainly the CIRNI-SAKAA that decreased from 37.3% to 27.0% (*χ*^2 ^= 1.57, P = 0.210) (Figure 2). Logistic regression analysis revealed that all of the above mentioned statistical results (with regards to significance) persisted after appropriate adjustment for *P. falciparum *density and age.

The prevalence of single nucleotide polymorphisms in *Pfdhfr *and *Pfdhps *is shown in Table 2. In *Pfdhfr*, only minor differences can be seen between the two cross sectional surveys where a strong trend for a decrease in the prevalence of the 108S wildtype is apparent (from 49.6% to 38.0%, *χ*^2 ^= 3.06, P = 0.080). For *Pfdhps*, as expected from the combined haplotype data, both a decrease of the 436/437SA wildtype (from 85.7% to 71.0%, *χ*^2 ^= 7.18, P = 0.007) and an increase in the mutant 436/437SG mutant type (from 56.4% to 68.5%, *χ*^2 ^= 3.46, P = 0.063) is evident. Furthermore, the prevalence of the 540 K wildtype is decreasing significantly from 92.1% to 71.6% (*χ*^2 ^= 16.66, P < 0.001).

**Table 2:** The prevalence of single nucleotide polymorphisms (SNP) at codon 51, 59 and 108 of *Pfdhfr *and codon 436/437 and 540 of *Pfdhps *in the two cross sectional surveys

*Pfdhfr *codon		Cross1		Cross2		P
			
		N	(%)	N	(%)	
51	N	43	32.3	33	27.8	0.169
	I	49	36.8	58	46.0	0.507
	Mixed (N/I)	41	30.8	33	26.2	
59	C	16	12.7	14	11.0	0.853
	R	66	52.4	69	54.3	0.828
	Mixed (C/R)	44	87.3	44	34.6	
108	S	14	11.0	8	6.2	0.080
	N	64	50.4	80	62.0	0.249
	Mixed (S/N)	49	38.6	41	31.8	

*Pfdhps *codon		N	(%)	N	(%)	

436/437	AA	3	2.4	1	0.8	0.746
	SA	50	39.7	37	29.8	0.007
	SG	15	11.9	35	28.2	0.063
	Mixed					
	AA/SA	2	1.6	1	0.8	
	SA/SG	56	44.4	45	36.3	
	AA/SA/SG	0	0	5	4.0	
540	K	80	63.5	73	54.5	< 0.001
	E	10	7.9	38	28.4	0.117
	Mixed (K/E)	36	28.6	23	17.2	

The data are given as total numbers and as percentages and including genotype infections where the identification of SNP/haplotypes for a few samples were not complete. Mixed: Prevalence of infections with mixed SNP for each codon. P: P-values of χ^2^-tests of prevalence comparisons between the two cross sectional surveys where the prevalence of infections of each SNP including mixed infections are set against prevalence of infections without the particular SNP.

Major differences in prevalence of infections with *Pfdhps *and combined *Pfdhfr*/*Pfdhps *mutations were found when comparing different age groups (i.e. 1–5, 6–14 and > 14 years), however only in the second survey (Table 3): The prevalence of quintuple mutant haplotype was significantly higher in children under fives compared to the older age groups (*χ*^2 ^= 6.04, P = 0.049) and a strong tendency for a higher frequency of *Pfdhps *double mutant haplotypes in children under fives compared to the older groups was seen (*χ*^2 ^= 5.33, P = 0.069).

**Table 3:** The prevalence of triple mutations in *Pfdhfr*, double mutations in *Pfdhps *and quintuple mutations in *Pfdhfr*/*Pfdhps *in the two cross sectional surveys according to age

	*Pfdhfr *triple MT		*Pfdhps double *MT		*Pfdhfr *and *Pfdhps* quintuple MT	
Age (years)	Cross1	Cross2	Cross1	Cross2	Cross1	Cross2

1–5	4/12 (33.3)	16/24 (66.7)	2/12 (16.7)	11/21 (52.4)	1/10 (10.0)	8/16 (50.0)
6–14	20/43 (46.5)	24/43 (55.8)	7/46 (15.2)	17/45 (37.8)	1/31 (3.2)	8/36 (22.2)
>14	22/44 (50.0)	18/42 (42.9)	7/46 (15.2)	12/49 (24.5)	4/34 (11.8)	7/37 (18.9)

P =	0.591	0.160	0.991	0.069	0.434	0.049

The donors are divided into 1–5 years, 6–14 years and above 14 years of age. Actual numbers of infections with *Pfdhfr *triple, *Pfdhps *double and *Pfdhfr*/*Pfdhps *quintuple mutations are shown out of total with the percentage of total in brackets. P: P-value of χ^2^-tests.

The samples analysis of the *Pfcrt *gene showed almost exclusively the occurrence of the mutant haplotype CVIET in both years (92.8% in survey 1 and 95.3% in survey 2; *χ*^2 ^= 0.74, P = 0.389), while the remaining samples were the wild type CVMNK haplotype.

## Discussion

In late 2002, Mozambique changed the treatment policy from CQ to SP/AQ against uncomplicated malaria. Prior to this change, the present study implemented SP alone as first line treatment in the study area and as a possible consequence, the study observed a rapid increase in frequency of double SGEAA-*Pfdhps *and quintuple CIRNI-SGEAA-*Pfdhfr/Pfdhps *mutant haplotypes at the community level just a year after. Prior to the introduction of SP the consumption of this drug was very low in the study area [19]. However, since the study did not measure the actual use of SP outside the health services, the study cannot rule out alternative explanations such as consumption of other antifolate drugs and for instance changes in migration. The triple mutant CIRNI-*Pfdhfr *haplotype, was highly prevalent prior to the change (46.5%), also observed in 2000 in Matola (50.9%), a peri-urban area within Maputo province [21]. Similarly, Raman *et al *observed a remarkable increase of the triple mutant haplotype in 13 sentinel sites stratified into three zones in Maputo province between 1999 and 2003, almost reaching 100% in 2003 [18], while Fernandes *et al *found 93% of the triple mutant haplotype in samples from children with uncomplicated malaria attending a Health Centre in Maputo city of Mozambique in 2004 [17].

This high frequency of *Pfdhfr *mutations before the actual implementation of SP/AQ as first-line drug in Mozambique may be due to the use of other chemically related drugs such as co-trimoxazole, which is commonly used in the study area for treating bacterial infections and as a preventive measure in HIV/AIDS patients (Dr Yunuss Assane, MoH, personal communication). While not shown *in vivo, in vitro *cross-resistance has been reported between co-trimoxazole and SP components [22]. More importantly, as suggested by Raman *et al*, since neighbouring KwaZulu-Natal province in South Africa implemented SP as early as 1988, the high frequency before SP implementation in Mozambique may be caused by SP drug pressure from the border region [18].

In the present study, the most notable change was the increase of the double mutant *Pfdhps *SGEAA haplotype from 13.5% to 34.8% over only one year. In the study by Raman *et al*, the frequency of the double mutant haplotype peaked in 2001 at 19–25% in all three zones followed by a remarkable and significant decrease in 2003 and 2004 to less than 10% [18], in sharp contrast to the findings by Fernandes *et al*, where the frequency was 51% in their 2004 study [17], presumably due to differences in sampling where the latter study was done on samples from symptomatic children attending a health centre. Such a difference in frequency in symptomatic cases versus asymptomatic carriers has likewise been shown in a study performed in Tanzania [23].

In the second survey of this study, the quintuple *Pfdhfr*/*Pfdhps *haplotype was more frequent in children under fives compared to the older age groups. Similar findings, but rather for *Pfdhfr *haplotypes alone was seen by Raman *et al *[18] and is most likely due to a stronger survival advantage of the mutant parasites in the younger age groups due to the more frequent treatments this group receive. It also pinpoints a potential pitfall when measuring prevalence of molecular markers of drug resistance in under-fives when these data are extrapolated to cover the population as a whole.

The high prevalence of these mutant haplotypes in the area as early as 2001 along with the findings by Fernandes *et al *[17] and Raman *et al *[18], questions the current efficacy of the first-line antimalarial drug in Mozambique, a combination of ART and SP. Results from *in vivo *study carried out in the same area in 2001 have described a relatively high adequate clinical response to SP after 14 days of follow-up at 82.7% [6]. However, the levels of treatment failures and RI and RII parasitological resistance increased when follow-up was extended to 28 days, indicating a decline in the efficacy of SP as early as 2001. Most likely, initially ART/SP was an efficient treatment against uncomplicated malaria in Mozambique, however the risk of clinical treatment failures could be expected to appear within a short span of years, since the benefits of ACT combinations are diminished by the inclusion of an ineffective partner drug, such as SP, where ART/SP may be equivalent to ART monotherapy. Despite that mutations in *Pfdhfr*/*Pfdhps *may not directly cause clinical ART/SP treatment failures, a high prevalence of SP resistance-related mutations may rather indicate a risk of emerging tolerance to ART endangering the whole ACT strategy in the region. Close and frequent monitoring of the efficacy of the drug combination should be performed as well as formulation of a strategy for alternative first line treatment.

## Conclusion

The high frequency of quintuple *Pfdhfr*/*Pfdhps *haplotypes found here as early as 2002 most likely cause ideal conditions for the development of ART tolerance in the *P. falciparum *populations and may even endanger the sensitivity to the second-line drug, Coartem.

## Authors' contributions

SE, PM, AMR, RT and MA conceived and designed the study. SE, FA, FXG-O and RT was responsible for the cross sectional sampling. SE performed the experiments and analyses with MA. SE, FXG-O, PM, FA, RT and MA participated in manuscript preparation. All authors read and approved the final manuscript.
